# Exercise capacity in relation to autoantibodies in systemic sclerosis patients

**DOI:** 10.1007/s00296-012-2541-y

**Published:** 2012-11-05

**Authors:** Fujiko Someya, Naoki Mugii, Tetsutarou Yahata, Takao Nakagawa

**Affiliations:** 1 School of Health Sciences, Kanazawa University, Kodatsuno 5-11-80, Kanazawa, 920-0942 Japan; 2 Division of Rehabilitation, Kanazawa University Hospital, Takaramachi 13-1, Kanazawa, 920-8640 Japan

**Keywords:** Systemic sclerosis, 6-Min walking test, Autoantibody, Lung function, Oxygen saturation

## Abstract

Autoantibodies have been detected in systemic sclerosis patients, and typical clinical features regarding organ involvement by each autoantibody have been reported. To reveal differences in exercise intolerance in patients with either anti-topoisomerase-I or anti-centromere antibodies, 53 systemic sclerosis patients were investigated retrospectively. A 6-min walking distance showed no significant differences (*P* = 0.090) between autoantibodies, while exercise-induced hypoxia during the 6-min walking test was significant in subjects with the anti-topoisomerase-I antibody (*P* = 0.033). The percent predicted of vital capacity, the diffusion capacity of the lung for carbon monoxide, and the modified Rodnan skin score were affected more in subjects with the anti-topoisomerase-I antibody than the anti-centromere antibody. The main parameter affecting the 6-min walking distance was the percent predicted of vital capacity for each autoantibody, and there was a significant positive relationship for all subjects (*R* ^*2*^ = 0.30, *P* < 0.0001). Exercise-induced hypoxia was also shown in the more affected subjects in the percent predicted of vital capacity and the diffusion capacity of the lung for carbon monoxide. Lung parameters were suggested to be more important factors determining exercise intolerance and induced hypoxia than detected autoantibodies.

## Introduction

Systemic sclerosis (SSc) is classified into two subsets, diffuse cutaneous SSc and limited cutaneous SSc, according to the extent of skin lesions. Some autoantibodies in sera found in SSc patients include anti-topoisomerase-I and anti-centromere antibodies [1–3]. The anti-topoisomerase-I antibody likely causes diffuse cutaneous SSc and a significant increased risk of lung and heart involvement. In contrast, the anti-centromere antibody causes limited cutaneous SSc and less organ involvement.

Exercise intolerance was also examined in SSc patients regarding organ dysfunction [4], and interstitial lung disease was suggested to be important in predicting mortality [5]. However, there are few studies regarding the role of autoantibodies on exercise capacity. The anti-topoisomerase-I antibody (anti-Sel-70 autoantibody) was reported to be a factor determining oxygen desaturation during the 6-min walking test (6MWT), where compared autoantibodies in the analysis were unclear [6].

The aim of this study is to reveal the role of autoantibodies on exercise capacity. Since possible organ involvement by each autoantibody is becoming clear, it must be established whether exercise intolerance is typically found in SSc with the anti-topoisomerase-I antibody or is caused by organ dysfunction despite autoantibody specificity.

## Methods

Fifty-three consecutive SSc patients with either anti-topoisomerase-I or anti-centromere antibodies who could perform the 6MWT were assigned to this study retrospectively (Table 1). Patients were referred as part of their routine evaluation for treatment. Evaluations were performed during their first visit to our facility from 2007 to 2011. The study was approved by the Ethics Committee of our facility. Oxygen saturation (SpO_2_) was monitored during the 6MWT. The SpO_2_ value was sufficiently high in all subjects (>95 %) at rest, while a decrease from baseline ≥4 % after the 6MWT was defined as exercise-induced hypoxia. Percent predicted of vital capacity (VC) and the diffusion capacity of the lung for carbon monoxide (D_LCO_) from spirometry were collected as pulmonary parameters. Skin involvement was evaluated by the modified Rodnan skin score.

**Table 1:** Characteristics of subjects

Parameters	Anti-topoisomerase-I antibody	Anti-centromere antibody	*P*
Sex (f/m)	32/5	15/1	0.65
Age (years)	54.3 ± 12.3	63.0 ± 8.9	0.0062
Duration of disease (years)	5.0 ± 5.1	10.9 ± 11.1	0.057
Subset (diffuse/limited)	30/7	0/16	NA
Vital capacity (% pred)	85.5 ± 22.9	110.6 ± 20.3	0.0004
D_LCO_ (% pred)	53.9 ± 16.8	68.2 ± 19.9	0.019
MRSS	14.6 ± 9.9	3.6 ± 4.3	<0.0001
6MWT distance (% pred)	85.7 ± 15.7	95.1 ± 18.8	0.090
Induced hypoxia/negative	20/17	3/13	0.033

Values are mean ± SD

*NA* not available, *MRSS* modified Rodnan skin score, *6MWT* 6-minute walking test

### Statistics

Walking distance was calculated as a percent of predicted values by the Enright formula [7] taking gender, age, height, and weight into consideration. Differences in evaluation values between the two autoantibodies were compared using the *t* test. The chi-square test was used for the comparison of sex distribution, subset of disease, and exercise-induced hypoxia. Simple linear regression between the 6MWT distance and parameter values was performed for each autoantibody. Statistical analyses were performed with JMP8.0 (SAS Institute, Cary, NC, USA). In all analyses, *P* < 0.05 was taken to indicate significance.

## Results

The anti-topoisomerase-I antibody was detected in 37 of 53 subjects and 30 of them were diagnosed with diffuse cutaneous SSc, whereas all 16 subjects with the anti-centromere antibody had limited cutaneous SSc (Table 1). There was no difference in sex distribution between the autoantibodies, though age was significantly higher and duration of disease after the onset of Raynaud’s phenomenon tended to be longer in subjects with the anti-centromere antibody. There was no significance in the 6MWT distance between autoantibodies (*P* = 0.090), while hypoxia induced by the 6MWT was significant in subjects with the anti-topoisomerase-I antibody (*P* = 0.033). Moreover, percent predicted of VC, D_LCO_, and the modified Rodnan skin score were more affected in subjects with the anti-topoisomerase-I antibody than the anti-centromere antibody.

**Fig. 1:**
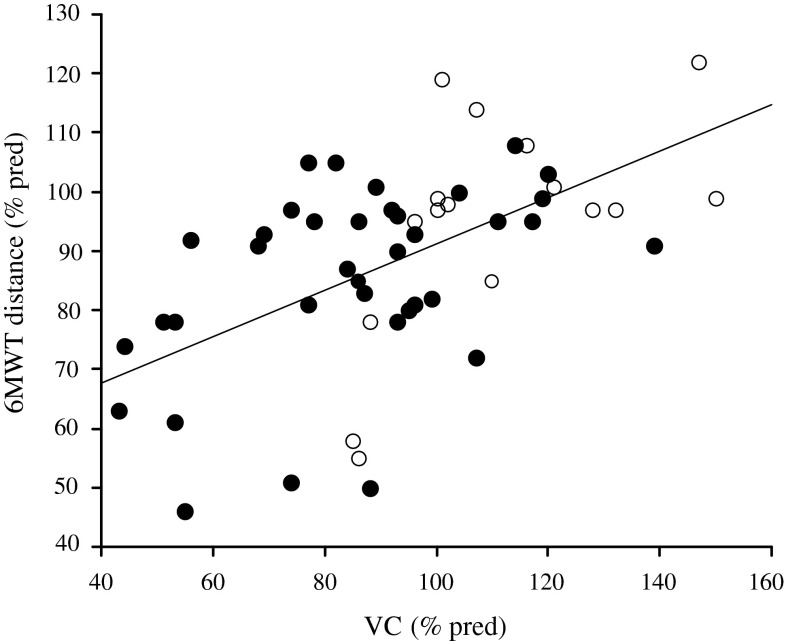
Relationship of percent predicted of vital capacity to the 6MWT distance. Closed *circles* represent the anti-topoisomerase-I antibody and open circles represent the anti-centromere antibody. The regression line is the 6MWT distance (% pred) = 53.8 + 0.37*VC (% pred) (*R* ^*2*^ = 0.30, *P* < 0.0001)

The only parameter affecting the 6MWT distance was percent predicted of VC for each autoantibody (Table 2). The regression line between the 6MWT distance and percent predicted of VC for all subjects represented a positive relationship (*R* ^*2*^ = 0.30, *P* < 0.0001) (Fig. 1). Induced hypoxia was shown in the more affected subjects in percent predicted of VC and D_LCO_, but not in age, duration of disease, or the 6MWT distance (Table 3).

**Table 2:** Simple linear regression analysis between the 6MWT distance and parameters

Parameters	Anti-topoisomerase-I antibody		Anti-centromere antibody	
	*R* ^*2*^	*P*	*R* ^*2*^	*P*
Vital capacity (% pred)	0.22	0.0034	0.33	0.020
D_LCO_ (% pred)	0.016	0.46	0.13	0.18
MRSS	0.086	0.079	0.044	0.44

*MRSS* the modified Rodnan skin score

**Table 3:** Effects of parameters on exercise-induced hypoxia

Parameters	Induced hypoxia	Negative response	*P*
Age (years)	57.3 ± 10.7	56.7 ± 13.0	0.85
Duration of disease (years)	6.0 ± 4.5	7.3 ± 9.7	0.51
Subset (diffuse/limited)	17/6	13/17	0.049
Vital capacity (% pred)	78.3 ± 21.2	104.3 ± 21.5	<0.0001
D_LCO_ (% pred)	45.4 ± 13.2	68.1 ± 16.4	<0.0001
MRSS	12.7 ± 9.8	10.3 ± 10.1	0.38
6MWT distance (% pred)	86.4 ± 19.4	90.1 ± 15.2	0.45

Values are mean ± SD

*MRSS* the modified Rodnan skin score, *6MWT* 6-minute walking test

## Discussion

In this study, as supported by previous studies [1, 3], lung and skin involvement was found in SSc with the anti-topoisomerase-I antibody more than that with the anti-centromere antibody. Moreover, the tendency of a longer duration from the onset of disease without severe organ dysfunction in subjects with the anti-centromere antibody was suggested by the rapid progress in lung involvement by the anti-topoisomerase-I antibody [8].

The main aim of this study was to define the limiting factors of exercise capacity. The distance of the 6MWT tended to be shorter in SSc with the anti-topoisomerase-I antibody than that with the anti-centromere antibody, but there was no significant difference despite distinguishable lung and skin involvement. However, simple linear regression analysis showed a clear relationship between the 6MWT distance and percent predicted of VC. These results suggested exercise intolerance was mainly caused by lung dysfunction, which was also shown in subjects with the anti-centromere antibody.

Exercise-induced hypoxia was more common in SSc with the anti-topoisomerase-I antibody than that with the anti-centromere antibody [6]. Since there were only three subjects with the anti-centromere antibody showing induced hypoxia, it was difficult to detect the affecting factors on induced hypoxia divided by each autoantibody. Lung involvement was significantly severe in subjects with induced hypoxia; however, skin involvement and/or exercise capacity did not affect oxygen saturation. Therefore, there remained the possibility that induced hypoxia was also caused by lung involvement and not by autoantibodies per se.

Other autoantibodies include anti-RNA polymerase, anti-U1-RNP, and anti-U3-RNP antibodies. As the anti-U1-RNP antibody is known to cause isolated pulmonary arterial hypertension [9], there is the possibility that a different relationship could exist between exercise capacity and examined parameters. This is because exercise capacity could also be reduced by pulmonary arterial hypertension [6, 10]. The distribution of autoantibodies in SSc has regional variety [3], and there were only two patients with the anti-U1-RNP antibody in this study. We excluded such a small number of cases and examined only two major autoantibodies. In the 53 subjects in this study, there was no relationship between the 6MWT distance and right ventricular systolic pressure (*R* ^*2*^ = 0.0013, *P* = 0.79), which may be the result of comparatively low pulmonary arterial pressures in these subjects.

Detection of autoantibodies would be beneficial to SSc patients for predictive prognosis concerning organ involvement. Even though the anti-centromere antibody has less of an effect on organs than that of the anti-topoisomerase-I antibody, organ involvement could not be avoided in disease of a long duration. Lung parameters were suggested to be important determinants of exercise intolerance and induced hypoxia in spite of whichever autoantibody was positive. In conclusion, careful examination of organ involvement is necessary regarding exercise capacity even after detection of autoantibodies.
